# Editorial: Archaeal Ribosomes: Biogenesis, Structure and Function

**DOI:** 10.3389/fmicb.2021.800052

**Published:** 2021-12-07

**Authors:** Sébastien Ferreira-Cerca, Anna La Teana, Paola Londei

**Affiliations:** ^1^Biochemistry III-Regensburg Center for Biochemistry, Institute for Biochemistry, Genetics and Microbiology, University of Regensburg, Regensburg, Germany; ^2^Department of Life and Environmental Science, New York-Marche Structural Biology Center (NY-MaSBiC), Polytechnic University of Marche, Ancona, Italy; ^3^Department Molecular Medicine, Policlinico Umberto I°Viale Regina, University of Rome Sapienza, Rome, Italy

**Keywords:** ribosome, archaea, translation, ribosome biogenesis, RNA modification

At the end of the 1970s, Carl Woese identified microorganisms that belong to a separate domain of life, the archaea (Fox et al., 1977; Albers et al., 2013). In the subsequent years, in-depth studies of the molecular and cellular biology of archaea, as well as of their physiology and environmental distribution, have revealed that archaea possess some very unique and distinctive biological traits (Albers et al., 2013; Tahon et al., 2021).

Such unique archaeal traits are also observed in fundamental cellular mechanisms like translation and ribosome biogenesis. Despite the universality of the translation process performed by the ribosome, significant molecular and functional similarities, as well as differences, underlying the mechanism of translation and ribosome's biology have been established across the tree of life.

Due to the central position of the translation machinery for gene expression and regulation, unraveling details of ribosome biogenesis and structure, as well as understanding its role in protein homeostasis is an emerging topic. Notably, and in contrast to bacteria and eukaryotes where a wealth of information is available, little is known as yet about the biogenesis and function of archaeal ribosomes.

The present Research Topic “*Archaeal Ribosomes: Biogenesis, Structure, and Function*” covers fundamental aspects of archaeal ribosome biology, from biogenesis to function.

In the first part of this Research Topic, three reviews and three original articles provide key insights into ribosome biogenesis in Archaea.

Londei and Ferreira-Cerca give an overview of ribosome biogenesis in archaea and highlight the similarities and differences with respect to the paradigm ribosome biogenesis pathways determined in bacterial and eukaryotic model organisms. Furthermore, Czekay and Kothe and Breuer et al. provide an in-depth summary on our knowledge of RNA-guided ribosomal RNA modifications, which require molecular machineries uniquely shared between archaea and eukaryotes, and discuss possible functional implications for ribosome biogenesis and function.

Ribosome biogenesis requires the action of trans-acting factors, known as ribosome biogenesis or assembly factors. However, little is known about putative archaeal ribosome biogenesis factors and their conservation across archaea. Birikmen et al. provide an in-depth bioinformatic analysis, and a state-of-the-art overview of ribosome biogenesis factors conservation across archaea and eukaryotes. Their study describes a core set of putative ribosome biogenesis factors widely conserved among archaea that may contribute to ribosome synthesis, although this is still to be proven experimentally. Notably, this study also reveals that the expansion of ribosome biogenesis factors characteristic of the eukaryotic pathway is not present in the Asgard archaea phylum which has been proposed to be more closely related to eukaryotes (Tahon et al., 2021). Altogether, this comprehensive study provides an important resource that will further help to functionally explore the archaeal ribosome biogenesis pathway and its evolution.

Detailed data on *in vivo* functional characterization of ribosome biogenesis in archaea remain relatively scarce. Using a recently developed gene repression system based on CRISPR technology (Stachler and Marchfelder, 2016), Schwarz et al. provide new insights into ribosomal RNA maturation and the *in vivo* role of the tRNA splicing endonuclease. The latter, in addition to its classical function in tRNA maturation, is also required for ribosomal RNA maturation *in vivo*, in agreement with recent *in vitro* data (Qi et al., 2020). This study further supports the functional requirement of precursor rRNA circularization, a specific characteristic of most archaeal ribosome biogenesis pathways analyzed thus far (Tang et al., 2002; Danan et al., 2012; Jüttner et al., 2020; Qi et al., 2020).

Finally, the study of Birkedal et al. also provides new insight into ribosome biogenesis diversity. Indeed, this work reveals a non-canonical 23S rRNA maturation pathway and describes the first known example of circularly permuted ribosomal RNA. This study also highlights the fact that ribosome biogenesis in archaea might be more diverse than so far anticipated.

Overall, there is still much to learn from the archaeal world, and much room for new surprising and exciting discoveries that will have key implications for our general understanding of ribosome biogenesis and evolution.

In the second part of this Research Topic, ribosome function is the focus of three reviews and two original research articles.

An emerging topic is the presence of transcription-translation coupling in archaea. Coupled transcription-translation has been proposed as a general mechanism of gene expression regulation in bacteria and archaea (Irastortza-Olaziregi and Amster-Choder, 2021); however, evidence for this coupling remains limited to a few archaeal species (French et al., 2007). Based on recent discoveries in bacteria and archaea, Weixlbaumer et al.'s perspective article explores the possible molecular basis of functional transcription-translation coupling in archaea. Furthermore, by using a systematic co-purification approach in *Haloferax volcanii*, Schramm et al. provide additional evidence for transcription-translation coupling in this cellular context, as well as additional insights into the interactions among translation initiation factors as well as among initiation factors and RNA polymerase components. Obviously, many future studies will be required to gain a better and wider understanding of the general prevalence of transcription-translation coupling in archaea, and on its molecular mechanism and functional advantages.

It has been previously established that translation initiation shares similar molecular features in archaea and eukaryotes. However, the structural and functional characteristics of assembly/disassembly of the pre-initiation and initiation complexes in archaea remain to be fully explored. Schmitt et al. provide an overview of the recent structural and functional advances on archaeal translation initiation. This summary is also complemented by additional insights from Lo Gullo et al. describing the release from the ribosome of the translation initiation factor aIF6 by the action of translation elongation factor EF-2.

Finally, De Lise et al. offer an overview on specific deviations from the standard ribosome decoding that have been identified in archaea.

In summary, the present Research Topic not only provides a state-of-the-art summary on our understanding of ribosome biogenesis and function in archaea, but also offers new perspectives and insights in this still emerging field, thus contributing to inspire and orient future research.

## Author Contributions

All authors listed have made a substantial, direct, and intellectual contribution to the work and approved it for publication.

## Conflict of Interest

The authors declare that the research was conducted in the absence of any commercial or financial relationships that could be construed as a potential conflict of interest.

## Publisher's Note

All claims expressed in this article are solely those of the authors and do not necessarily represent those of their affiliated organizations, or those of the publisher, the editors and the reviewers. Any product that may be evaluated in this article, or claim that may be made by its manufacturer, is not guaranteed or endorsed by the publisher.
